# A Potential Spatial Working Memory Training Task to Improve Both Episodic Memory and Fluid Intelligence

**DOI:** 10.1371/journal.pone.0050431

**Published:** 2012-11-28

**Authors:** Sarah R. Rudebeck, Daniel Bor, Angharad Ormond, Jill X. O’Reilly, Andy C. H. Lee

**Affiliations:** 1 Department of Experimental Psychology, University of Oxford, Oxford, United Kingdom; 2 Sackler Centre for Consciousness Science, University of Sussex, Brighton, United Kingdom; 3 Department of Informatics, University of Sussex, Brighton, United Kingdom; 4 Oxford Functional Magnetic Resonance Imaging of the Brain Centre, University of Oxford, Oxford, United Kingdom; 5 Department of Psychology (Scarborough), University of Toronto, Toronto, Ontario, Canada; 6 Rotman Research Institute, Baycrest Centre for Geriatric Care, Toronto, Ontario, Canada; University of California, San Francisco, United States of America

## Abstract

One current challenge in cognitive training is to create a training regime that benefits multiple cognitive domains, including episodic memory, without relying on a large battery of tasks, which can be time-consuming and difficult to learn. By giving careful consideration to the neural correlates underlying episodic and working memory, we devised a computerized working memory training task in which neurologically healthy participants were required to monitor and detect repetitions in two streams of spatial information (spatial location and scene identity) presented simultaneously (i.e. a dual n-back paradigm). Participants’ episodic memory abilities were assessed before and after training using two object and scene recognition memory tasks incorporating memory confidence judgments. Furthermore, to determine the generalizability of the effects of training, we also assessed fluid intelligence using a matrix reasoning task. By examining the difference between pre- and post-training performance (i.e. gain scores), we found that the trainers, compared to non-trainers, exhibited a significant improvement in fluid intelligence after 20 days. Interestingly, pre-training fluid intelligence performance, but not training task improvement, was a significant predictor of post-training fluid intelligence improvement, with lower pre-training fluid intelligence associated with greater post-training gain. Crucially, trainers who improved the most on the training task also showed an improvement in recognition memory as captured by d-prime scores and estimates of recollection and familiarity memory. Training task improvement was a significant predictor of gains in recognition and familiarity memory performance, with greater training improvement leading to more marked gains. In contrast, lower pre-training recollection memory scores, and not training task improvement, led to greater recollection memory performance after training. Our findings demonstrate that practice on a single working memory task can potentially improve aspects of both episodic memory and fluid intelligence, and that an extensive training regime with multiple tasks may not be necessary.

## Introduction

Long-term memory for personally experienced events, also known as episodic memory (EM), is a vital capacity that underlies many of our everyday functions. Given the importance of EM and the fact that EM is vulnerable to neuronal cell loss as a result of healthy ageing, traumatic brain injury or dementia [1], [2], [3], [4], [5], any cognitive training regime that enhances EM performance could potentially be of significant benefit to a wide range of people.

A common approach to EM training is cognitive ability training, which concentrates on the cognitive abilities that support EM [6]. The logic behind this approach is that improvement on a training task should transfer successfully to other domains/tasks, provided that these tasks rely on overlapping cognitive abilities, or common neural systems [7]. One stream of EM ability training research has targeted prefrontal cortex (PFC)-dependent executive functions in light of the important contribution the PFC makes to EM [8]. Of particular interest is working memory (WM) given the proposed interaction between WM and EM processes [9]. Surprisingly, the effects of working memory training on episodic memory have, to date, been mixed. Many studies have failed to observe any clear benefits of WM training on EM [10], [11], [12], [13] and when potential EM improvements have been reported, these rely on a large battery of training tasks [14], [15], which can be time-consuming and difficult to learn.

One possible reason why WM training has not been consistently associated with EM improvement is that previous research has not fully considered the neural substrates that underlie WM and EM. Medial temporal lobe (MTL) structures, such as the hippocampus, have been traditionally considered to subserve EM and not WM [16]. There is increasing evidence, however, that the MTL also supports WM, but only for specific types of information (i.e. novel, spatial/relational information) [17], [18], [19], [20], [21]. It is possible, therefore, that training on a WM task that recruits MTL structures will increase the likelihood of EM improvement. Notably, previous training studies have typically employed WM training tasks that do not involve the kinds of information associated with MTL involvement. A second significant limitation of existing EM training research is that previous investigations have generally assessed EM using standard neuropsychological tests or basic memory paradigms (e.g. recalling a limited word list). These tasks may not be sufficiently sensitive to detect subtle changes in EM ability and, critically, provide limited insight into how cognitive training may differentially affect different types of EM processes, for example, recollection (‘remembering’) and familiarity (‘feeling of knowing’) recognition memory. The latter is an important issue since there is evidence to suggest that different EM processes such as recollection and familiarity are subserved by different neural substrates [22], [23] and thus, the effectiveness of a single training paradigm to provide general improvements in EM is likely to be determined by the brain structures that the training task recruits. For instance, considering MTL structures, one viewpoint suggests that recollective memory is dependent on the hippocampus whereas familiarity is subserved by the cortex adjacent to this structure, more specifically the perirhinal cortex [22], [23] (see, however, [16]). According to this view, therefore, a training task that primarily targets the hippocampus may predominantly impact recollective but not familiarity memory.

**Figure 1:**
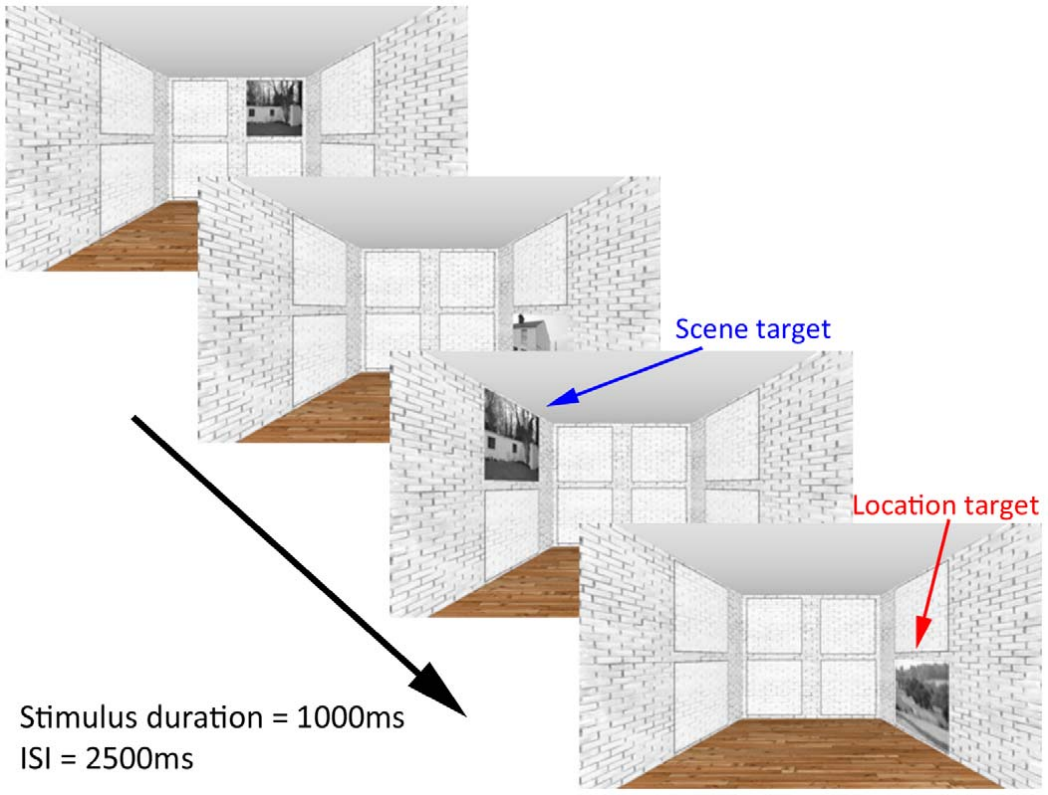
Schematic diagram of training paradigm. In this example, participants had to detect spatial locations and scene images that were identical to those presented two trials earlier (2-back task). The N-back requirement started at 1 and varied with performance across training.

To address these issues and investigate whether a single WM task could benefit EM, we designed a novel adaptive spatial WM training task in which participants monitored real-world scenes presented across 8 picture frames located in a three-dimensional (3D) room (Figure 1). Our decision to emphasize spatial processing was motivated by a recent study in which we demonstrated that increased WM demand for complex spatial information led to greater PFC and MTL activity [19]. Participants’ EM abilities were assessed before and after training using one object and one scene recognition memory task incorporating memory confidence judgments. These two tasks allowed us to (1) investigate recognition performance with regards to hits and false alarms (i.e. d-prime, d′); (2) acquire quantitative measures of recollection and familiarity by taking into account confidence judgments, which may be more sensitive to changes in recognition memory ability (see Methods); and (3) assess recognition memory using two different types of stimuli to determine whether the use of spatial stimuli for the training task would lead to improvements in recognition memory involving other types of stimuli (i.e. objects). To determine the generalizability of the effects of training beyond recognition memory, we also assessed fluid intelligence (G*f*) using a matrix reasoning task (Bochumer Matrices Test, BOMAT) [24]. The use of this test was motivated by recent work that has demonstrated that WM training can benefit G*f* [25]. We predicted, therefore, that our spatial WM training task would at least lead to significant improvements in G*f* and potentially, in recognition memory performance as well, as captured by some or all of the different performance measures used.

## Methods

### Participants

56 neurologically healthy right-handed young volunteers who were not on any psychoactive medication were recruited from the Oxfordshire (UK) area, with one half forming the training group, and the other half a control group. One training volunteer was excluded due to illness that interfered with participation. For the remaining participants, there were no significant differences (all t(53)<0.7, p>0.5) between the training group and controls in terms of age, sex, education, verbal IQ as measured by the National Adult Reading test (NART) [26] or depression as measured by the Beck Depression Inventory II (BDI) [27] (Table 1). To investigate any potential effects of differences in training task gain (i.e. quality of training), we also divided the training participants into a high gain (HG) group (training task gain scores above the group median, n = 14) and a low gain (LG) group (training task gain scores below the group median, n = 13). There were no significant differences on any of the descriptive factors (all F(2, 52)<1.4, p>0.2) between the controls, HG trainers and LG trainers (Table 1). This study was approved by the Central University Research Ethics Committee at the University of Oxford, and all participants gave informed written consent prior to participation.

**Table 1:** Demographic details of the training group and control participants considered as a single group, and divided into two groups according to training task gain (HG: high gain; LG: low gain; BDI: Beck Depression Inventory II; NART: National Adult Reading Test).

	All trainers Mean (S.D.)	Controls Mean (S.D.)	HG Trainers Mean (S.D.)	LG Trainers Mean (S.D.)
**Female: Male**	13∶14	14∶14	6∶8	7∶6
**Age/years**	25.36 (4.45)	25.49 (4.68)	26.74 (4.41)	23.88 (4.15)
**Education/years**	16.63 (2.87)	16.14 (2.51)	16.57 (2.31)	16.69 (3.47)
**BDI score**	2.89 (3.29)	3.18 (3.85)	2.71 (3.15)	3.08 (3.55)
**NART score**	36.33 (8.96)	37.00 (7.95)	34.79 (7.36)	38.00 (10.46)

### Behavioral Procedure

The training participants were asked to practice on the training task for 20 minutes a day on their personal computers, 5 days a week for 4 weeks. The duration of training (20 days) was determined on the basis of previous research that has demonstrated that training-related cognitive improvements are time-dependent, with large effects observable after a period of about 20 days [25]. To track training, a performance log file was automatically generated after each training session and given to the investigators by the participants. All trainers were able to provide a log file for every training day other than one participant whose log files for 7 days (spread throughout the 20 days of training) were unfortunately irretrievable due to computer technical difficulties. A pre-training assessment session took place the day before training began and the post-training session no more than 2 days after training was completed. The control group completed the same evaluation sessions as the trainers 28 days apart, but did not undertake any cognitive training.

### Assessment Sessions

In the pre- and post-training sessions, all participants completed the training task, the BOMAT, and two recognition tests. The training and recognition tasks were administered on a 15″ laptop computer, whereas the BOMAT was administered on paper. Different versions of each task were used across the two assessment sessions, and within each session, the same tasks were administered in the same order across all participants. All pre- and post-training assessment scores are provided in Table 2.

**Table 2:** The pre- (1) and post-training (2) assessment scores for the training task, BOMAT, and recognition memory tests (average d′, recollection and familiarity scores, as well as individual scores for the object and scene tasks) (HG: high gain; LG: low gain).

Task	Session	All trainersMean (S.D.)	ControlsMean (S.D.)	HG trainersMean (S.D.)	LG TrainersMean (S.D.)
**Training task**	1	1.53 (0.28)	1.65 (0.38)	1.57 (0.31)	1.49 (0.27)
	2	2.86 (0.93)	1.69 (0.43)	3.49 (0.83)	2.19 (0.42)
**BOMAT**	1	7.56 (2.45)	7.50 (2.32)	8.36 (2.41)	6.69 (2.29)
	2	9.52 (2.03)	7.75 (2.53)	9.93 (2.13)	9.08 (1.89)
**Average d′**	1	1.28 (0.31)	1.20 (0.34)	1.31 (0.31)	1.25 (0.31)
	2	1.21 (0.43)	1.02 (0.42)	1.45 (0.35)	0.96 (0.35)
**Average recollection**	1	0.25 (0.13)	0.22 (0.12)	0.23 (0.13)	0.27 (0.12)
	2	0.24 (0.14)	0.14 (0.10)	0.28 (0.15)	0.19 (0.12)
**Average familiarity**	1	0.84 (0.38)	0.87 (0.32)	0.90 (0.29)	0.77 (0.46)
	2	0.86 (0.45)	0.79 (0.34)	1.09 (0.29)	0.62 (0.48)
**Object d′**	1	1.42 (0.37)	1.26 (0.39)	1.45(0.42)	1.38 (0.33)
	2	1.29 (0.53)	1.01 (0.43)	1.54 (0.53)	1.02 (0.37)
**Object recollection**	1	0.28 (0.15)	0.23 (0.14)	0.26 (0.16)	0.30 (0.15)
	2	0.27 (0.16)	0.15 (0.12)	0.32 (0.16)	0.22 (0.14)
**Object familiarity**	1	0.94 (0.47)	0.95 (0.40)	1.02 (0.38)	0.85 (0.55)
	2	0.88 (0.60)	0.80 (0.38)	1.13 (0.48)	0.62 (0.61)
**Scene d′**	1	1.15 (0.33)	1.14 (0.36)	1.17 (0.32)	1.13 (0.35)
	2	1.10 (0.46)	1.03 (0.45)	1.28 (0.47)	0.90 (0.37)
**Scene recollection**	1	0.23 (0.11)	0.21 (0.12)	0.21 (0.11)	0.25 (0.12)
	2	0.19 (0.14)	0.14 (0.09)	0.22 (0.16)	0.16 (0.11)
**Scene familiarity**	1	0.74 (0.34)	0.79 (0.34)	0.79 (0.25)	0.69 (0.42)
	2	0.82 (0.47)	0.79 (0.35)	1.01 (0.46)	0.62 (0.40)

### Training Task

We developed a dual N-back spatial working memory task in Presentation (Neurobehavioral Systems Inc.). Participants were presented with a 3D room containing 8 picture frames (Figure 1). Images of real-world scenes were presented one at a time in these frames (duration 1000 ms, inter-stimulus interval 2500 ms) and participants were asked to monitor the real-world scenes as well as the locations in which they were presented. The task started as a 1-back task on the first block of training in the first training session, and was ratcheted to N-back in subsequent blocks according to the participant’s performance. An ‘S’ key response was required when a scene image was repeated (2 successive identical scenes for 1-back; 2 identical scenes separated by a different image for 2-back, and so forth) and an ‘L’ when a picture frame location was repeated (2 scenes presented successively in the same location for 1-back; 2 scenes presented in the same location, separated by a different location, for 2-back, and so forth). Each training session comprised 12 blocks. 30 scenes were presented in each block and of these 6 were scene targets, 6 were location targets and 2 were both scene and location targets. If the participant made fewer than 3 errors in both scene and location modalities within a block, the level of N increased by one in the next block. If more than 5 errors were made the level of N was decreased by 1 (minimum 1-back) and in all other cases the level of N remained the same.

The scene images consisted of 440 unfamiliar grayscale photographs of indoor and outdoor scenes, which did not contain people, objects, or words. These scenes were specifically picked so that they could not be easily encoded verbally. The N-back training task was quasi randomized so that in each training session a scene was only selected once by the program.

### Bochumer Matrices Test (BOMAT) – advanced Short Version

The BOMAT is a non-verbal neuropsychological test of G*f* [24]. In each trial a 5×3 matrix of patterns is presented with one empty field in the matrix. The participant must decide which pattern out of 6 options completes the matrix. In the advanced short version, there are 29 successive matrices to complete. Due to time restrictions and the possibility of ceiling effects associated with some G*f* tests, participants were given 10 minutes to complete as many patterns as they could in each assessment session (for a similar procedure see [25]). The number of correct responses during this time served as a measure of G*f*. Versions A and B of the BOMAT were presented in the pre- and post-test sessions, respectively.

### Recognition Memory Tasks

One scene and one object recognition task were administered in each assessment session. In both sessions, the participants were aware that these tasks were designed to assess their recognition memory. The procedure for each task was identical across both sessions, although different stimuli were used. Each scene task involved 240 grayscale photographs of unfamiliar indoor and outdoor scenes, which did not contain people, objects, or words. These scenes were different to those used in the training task. For each object test, 240 grayscale photos of everyday objects were used. In both tests the stimuli were split into 120 items for an encoding phase and 120 foil items, which were presented with the encoding items for a test phase. In the encoding phase, participants were presented with individual images and asked whether each scene was indoor or outdoor, or if each object could fit in a shoebox. Following 20 min, during which filler tasks unrelated to the recognition memory paradigm were completed (e.g. the NART and BDI), the test phase took place. Participants were shown the encoding items intermixed randomly with the foils and asked to make a recognition judgment for each item using a 6-point confidence scale (“1” = confident item is new; “6” = confident item is old).

A d-prime (d′) score (z(P(hits) – z(P(false alarms)) was calculated for each task as a general measure of performance. To take confidence ratings into account, ROC curves were derived by plotting P(hits) vs. P(false alarms) starting at the most confident response level (hits = P(6|old); false alarms = P(6|new)), and then cumulatively at subsequent confidence levels (hits = P(6|old)+P(5|old); false alarms = P(6|new)+P(5|new), etc). The dual process signal detection (DPSD) model (for details see [28], [29]) was fit to this data by using a Microsoft Excel Solver that implements a sum of squares search algorithm to obtain estimates of recollection and familiarity for each participant. In brief, the DPSD model assumes that recognition memory consists of independent recollection and familiarity components. According to this model, recollection is a threshold process and thus, can either fail or lead to the recall of varying amounts of qualitative information (e.g. when and where a previously encountered item was seen before). Thus, high confidence hits (e.g. 6) are believed to reflect recollective memory. On the other hand, recognition memory based on familiarity occurs when recollection fails, and is suggested to reflect a signal detection process in which studied and new items possess distinct but overlapping Gaussian distributions of memory strength.

### Data Analyses

Analyses were conducted using IBM SPSS software. The performance measures from the two recognition memory scores were considered collectively by averaging across the two tasks to create composite scores (i.e. average d′, average recollection, average familiarity) as well as separately (i.e. object d′, object recollection, object familiarity, scene d′, scene recollection, scene familiarity). To determine whether there were any pre-existing differences between the participant groups, the pre-training assessment scores of the controls and trainers were compared using two-tailed independent sample t-tests (when the trainers were considered as a single group) and one-way analyses of variance (ANOVA; when the trainers were median-split according to training gain into HG and LG groups). To investigate any changes in performance after training, gain scores (post- minus pre-training score) were calculated for all tasks. Two-tailed independent sample t-tests were first used to explore any differences between all the trainers and the control group. When the trainers were divided into HG and LG groups, one-way ANOVAs were used to contrast these two groups with the controls. Since only three means were involved (and thus, Family Wise Error rate = α), we used three 2-tailed linear contrasts (HG vs. LG, HG vs. controls, LG vs. controls) to explore these ANOVAs further [30]. Given the unequal sample sizes following median-split of the trainers, we paid particular attention to homogeneity of variance across groups. When this was violated as indicated by the Levene statistic, the Welch procedure was used and subsequent linear contrasts were adjusted. Finally, we explored whether any BOMAT and recognition memory improvements in the trainers were related to (1) the degree of improvement on the training task; and (2) the trainers’ pre-training abilities (as measured by pre-training assessment). To achieve this, we carried out a series of multiple regression analyses in the trainers with the transfer task gain score as the dependent variable (i.e. BOMAT score or one of the three composite recognition memory scores), and the corresponding pre-training score and training task gain score as the independent variables.

## Results

### Pre-training Scores

Irrespective of whether the trainers were considered as a single group, or as two groups according to training task gain (HG vs. LG), there were no significant differences in pre-training scores between the trainers and controls on any of the tasks administered (see Table 3). Thus, any significant differences between groups in gain scores cannot be attributed to pre-training differences.

**Table 3:** Statistical comparison of pre-training assessment scores (HG: high gain; LG: low gain).

Task	All trainers vs. controls		HG trainers vs. LG trainers vs. controls	
	t(53)	P	F(2, 52)	p
**Training task**	1.33	0.19	1.05	0.36
**BOMAT**	0.09	0.93	1.72	0.19
**Average d′**	0.98	0.33	0.57	0.57
**Average recollection**	1.15	0.26	1.01	0.37
**Average familiarity**	0.32	0.75	0.54	0.59
**Object d′**	1.55	0.13	1.28	0.29
**Object recollection**	1.33	0.19	1.20	0.31
**Object familiarity**	0.08	0.94	0.50	0.61
**Scene d′**	0.12	0.90	0.07	0.93
**Scene recollection**	0.74	0.47	0.57	0.57
**Scene familiarity**	0.56	0.58	0.45	0.64

### Gain Scores

**Figure 2:**
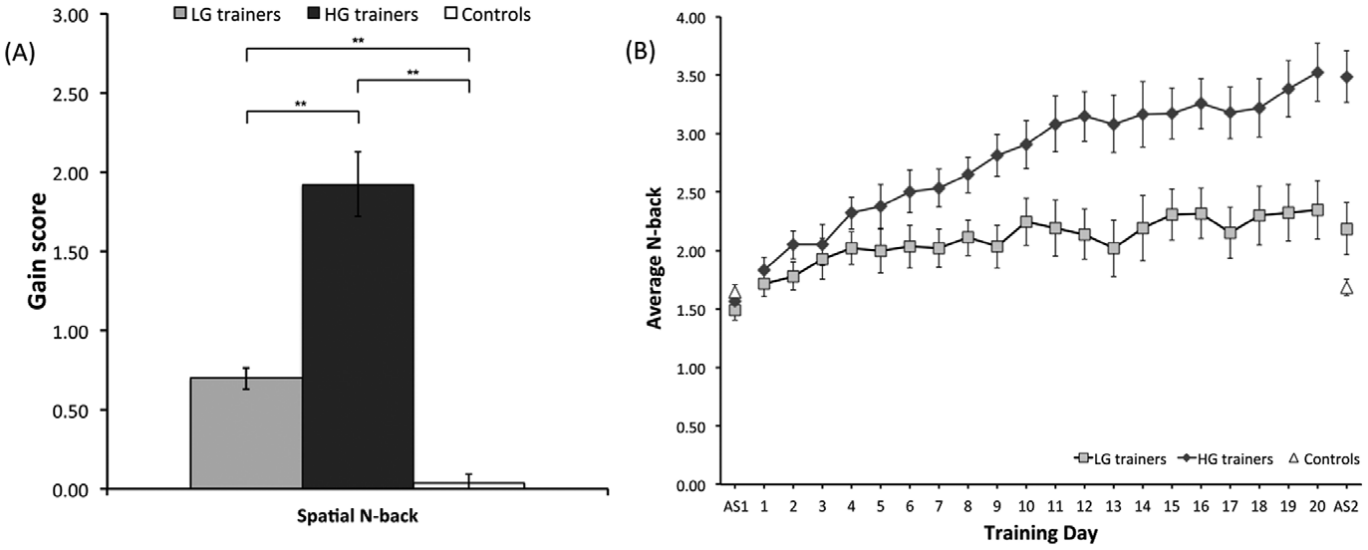
Mean gain scores (±S.E.) on the training task (A) and change in performance across 20 days of training for the LG and HG groups (B). The mean scores at the first (AS1) and second (AS2) assessments are also shown. ** p<0.001.

As expected, the training group possessed significantly greater gain scores on the training task compared to the control group (t(53) = 7.59; p<0.0001). When the training group was median split into an HG group and a LG group, a one-way ANOVA revealed a significant effect of group (Welch F′′(2, 25.10) = 53.80, p<0.0001) (Figure 2). There was a significant difference between the HG and LG groups (t(15.85) = 5.67, p<0.0001, Cohen’s d = 2.15), the HG group and controls (t(15.30) = 8.83, p<0.0001, Cohen’s d = 3.21), and the LG group and controls (t(29.85) = 7.24, p<0.0001, Cohen’s d = 2.32). The improvement in performance of both HG and LG groups over the 20 days of training could be explained by a linear function (HG: R^2^ = 0.93, F(1, 18) = 253.05, p<0.0001; LG: R^2^ = 0.79, F(1, 18) = 68.59, p<0.0001).

**Figure 3:**
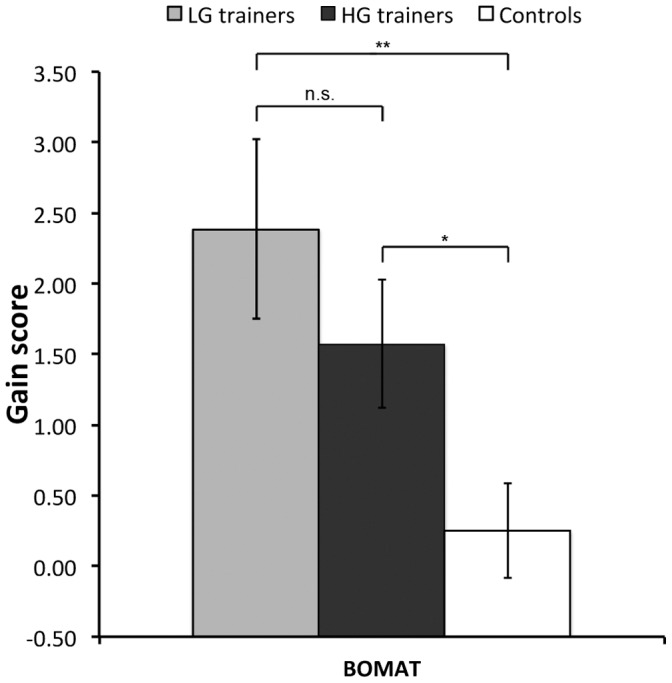
Mean gain scores (±S.E.) on the BOMAT. **p<0.001; *p = 0.05; n.s. = not significant.

Training led to significant improvement on the BOMAT. Overall, the trainers made a significantly greater improvement on this test in comparison to controls (t(53) = 3.14, p = 0.003). A one-way ANOVA also revealed a significant effect of group when the trainers were median-split (F(2, 52) = 5.50, p = 0.007), with a significant difference between the LG group and controls (t(52) = 3.15, p = 0.003, Cohen’s d = 0.99), as well as between the HG group and controls (t(52) = 2.00, p = 0.05, Cohen’s d = 0.71). Although there was a numerical difference between the HG and LG trainers, this was not statistically significant (t(52) = 1.05, p = 0.3, Cohen’s d = 0.40) (Figure 3). A multiple regression analysis in all the trainers revealed that pre-training BOMAT performance and training task gain explained a significant proportion of the variance in BOMAT gain (R^2^ = 0.40, F(2, 24) = 7.85, p = 0.02). It was found that while the pre-training BOMAT score was a significant predictor of BOMAT gain after training, with lower pre-training scores predicting greater post-training gain (β = −0.66, p = 0.001), training task gain was not a significant predictor (β = 0.19, p = 0.3).

**Figure 4:**
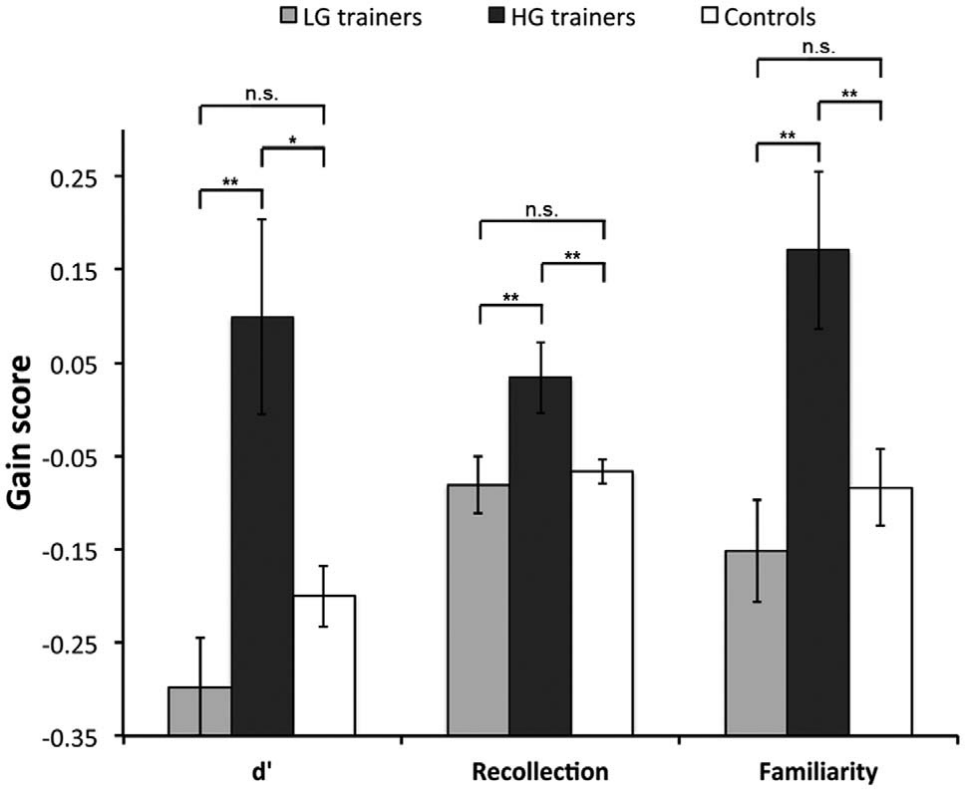
Mean gain scores (±S.E.) on the EM composite scores. **p<0.001; *p<0.05; n.s. = not significant.

There was a significant positive correlation between the gain scores on all measures of the two recognition tasks (d’ gain: r = 0.55, p<0.0001; recollection gain: r = 0.61, p<0.0001; familiarity gain: r = 0.34, p = 0.01). Although a number of negative gain scores were observed due to the second assessment tasks being more difficult than those in the first session, this does not affect the interpretation of our findings as all participants received identical tests and it is the differences in gain scores between groups that are critical. We found no significant difference in gain on any of the composite scores between the trainers, when considered as a single group, and non-trainers (all t(52)<1.7, p≥0.1). When HG and LG trainers were considered separately, however, striking differences emerged (Figure 4). There was a significant effect of group on all three composite scores (d′: Welch F′′(2, 24.77) = 5.87, p = 0.008, recollection and familiarity: both F(2, 52)>5.9, p≤0.005), and for each composite score, there was a significant difference between the HG and control groups (d′: t(16.87) = 2.46, p = 0.03, Cohen’s d = 0.95; recollection and familiarity: both t(52)>3.0, p≤0.004, Cohen’s d≥0.9), and the HG and LG groups (d′: t(18.97) = 3.40, p = 0.003, Cohen’s d = 1.29; recollection and familiarity both t(52)>2.9, p≤0.005, Cohen’s d≥0.9). There were no significant differences between the LG trainers and controls on any of the recognition composite scores (d′: t(26.18) = 1.86, p = 0.08, Cohen’s d = 0.49; recollection and familiarity: both t(52)<1.0, p≥0.3, Cohen’s d≤0.3).

To explore the recognition data further, we also considered the object and scene tasks separately. One-way ANOVAs revealed a significant effect of group on d’ gain for both tasks (scene: Welch F”(2, 27.07) = 3.55, p = 0.04; object: F(2, 52) = 10.30, p<0.0001), familiarity gain for both tasks (both F(2, 52)>3.2, p<0.05), and recollection gain for the object task (F(2, 52) = 6.44, p = 0.003), with a trend for recollection gain on the scene task (F(2, 52) = 2.98, p = 0.060). There was a significant difference between the HG group and controls on all three performance measures for the object recognition task (all t(52)>2.6, p≤0.01, Cohen’s d≥0.9 ), as well as a significant difference between the HG and LG trainers (all t(52)>2.9, p≤0.005, Cohen’s d≥0.9). On the scene task, there was a significant difference between the HG and LG groups on d’ gain (t(15.25) = 2.30, p = 0.04, Cohen’s d = 0.87), recollection gain and familiarity gain (both t(52)>2.1, p≤0.03, Cohen’s d≥0.7), a significant difference between the HG group and controls on recollection gain and familiarity gain (both t(52)>2.1, p<0.04, Cohen’s d≥0.6), but not between the HG trainers and controls on d’ gain (t(16.79) = 1.45, p = 0.17, Cohen’s d = 0.52). On both scene and object tasks, there were no significant differences between the LG trainers and controls on any of the recognition measures (Scene d′: t(38.05) = 1.79, p = 0.08; all others t(52)<1.3, p≥0.2; Cohen’s d≤0.5 ).

Lastly, to explore the relationship between training task gain, pre-training performance and recognition memory improvement, we conducted three separate multiple regression analyses in the trainers using the average d′, average recollection and average familiarity scores. These showed that a significant proportion of the variance in gain on each of these three composite measures could be explained by the trainers’ corresponding pre-training performance and training task gain (d′: R^2^ = 0.25, F(2, 24) = 3.95, p = 0.03; recollection: R^2^ = 0.28, F(2, 24) = 4.55, p = 0.02; familiarity: R^2^ = 0.23, F(2, 24) = 3.56, p = 0.04). For both d′ and familiarity, training task gain was a significant positive predictor of improvement (both ß>0.48, p<0.02), whereas pre-training performance was not a significant predictor (both −0.2<ß<0, p>0.4). In contrast, pre-training performance (ß = −0.47, p = 0.01), but not training task gain (ß = 0.29, p = 0.1), was a significant predictor of improvement in recollection, with lower pre-training recollection scores leading to greater recollection gain scores after training.

## Discussion

To our knowledge, we have demonstrated for the first time that an extensive cognitive ability training regime incorporating multiple tasks is not necessary to improve episodic memory (EM), as measured by two recognition memory tasks in the current study. We have shown instead that practice on only a single intensive, spatial working memory task can potentially enhance both long-term mnemonic and fluid intelligence abilities.

The present investigation contrasts with recent studies in which WM training transfer effects to EM have been absent or weak [10], [11], [12], [13]. Although successful transfer between a single WM training task and EM performance has not, to our knowledge, been demonstrated prior to our study, there are a number of theoretical reasons why one would expect WM training to benefit EM. First, PFC-dependent executive functions are suggested to facilitate successful EM encoding and retrieval [31], [32], [33], [34]. Thus, it is not surprising that prolonged practice on an executive task such as WM may aid EM processing. At a more specific level, WM processes have been suggested to interact with EM. For example, the ability to maintain information in WM may have an impact on EM encoding and retrieval [9], [35]. Finally, it has been suggested that the neural mechanisms underlying WM and EM may not be as distinct as previously thought. Traditionally, the MTL has been associated with EM but not WM processing, whereas the lateral PFC has been suggested to be critical for WM and not EM [16], [36]. This idea has, however, been challenged recently [36]. Contrary to early reports that patients with MTL damage demonstrate intact WM, recent work has shown that patients with MTL lesions do exhibit significant deficits when specific types of WM tasks are administered, in particular those that utilize stimuli that cannot be verbalized easily [37] or place a significant demand on relational/spatial processing [38], [39]. Crucially, comparable WM tasks, including those using delayed-match-to-sample and n-back paradigms, have been associated with MTL involvement in functional neuroimaging studies [18], [19], [20], with stimulus novelty also being a critical factor [17], [21]. Moreover, the degree of MTL activity during the maintenance of items in WM may be predictive of subsequent EM success for the same items [40].

In considering the overlapping neural correlates of WM and EM, we suggest that one critical factor in determining the transfer success of WM practice to EM is the type of stimuli used during training. Since the MTL is not involved in all forms of WM and complex spatial/relational processing has been implicated as one critical factor [19], [20], [38], our participants were required to not only monitor 8 spatial locations within a 3D room, but also a large collection of 440 unique spatial scenes (besides those used as repetitions, no image was used more than once in each training session). This contrasts with previous studies in which training tasks have taxed WM using relatively limited pools of 2D spatial locations, colors, digits, letters, or animal pictures, therefore leading to more frequent stimulus repetition throughout training.

Our observation of improved *Gf* (as measured by the BOMAT) following training supports previous work that has found G*f* improvement following WM training [25], [41] (see, however, [13], [42]). Adaptive WM training tasks may induce G*f* improvements since they require a wide range of executive processes, which are closely associated with G*f*. These include placing, updating and removing items in WM, inhibiting irrelevant information, monitoring performance, binding (e.g. combining a scene and location), as well as managing two complex goals simultaneously. In addition, the n-back task, dual task processing, binding/chunking, as well as G*f* based tasks, all robustly activate a common neural network across the PFC and parietal cortex [43], [44], [45]. Importantly, as noted above, many of these executive processes may also contribute to EM performance [31], [32], [33], [34], therefore explaining why improvements on the current training task may generalize to both G*f* and EM.

Besides the need for the training and transfer tasks to be mediated by overlapping neural substrates [7], there are other factors that are likely to influence the extent to which improvement on a training paradigm translates to better performance on other cognitive tasks. For instance, it has been proposed that the degree of training improvement is critical, with recent evidence suggesting that larger transfer effects are likely to occur following greater improvement on WM training [46], [47]. In addition to this, there has been some debate over whether the effects of cognitive training are restricted to individuals within a limited range of cognitive abilities [25], with the possibility that individuals possessing a lower pre-training ability level are more likely to benefit from training. The present data provide supporting evidence that both of these factors can influence the effectiveness of training and moreover, suggest that their impact may vary across different types of cognitive processes/tasks. We found that only the HG, and not LG, trainers demonstrated a significant benefit to recognition memory performance. This indicates that, broadly speaking, a certain degree of training improvement in WM for spatial information is necessary before a successful transfer effect to EM can be observed. In contrast, however, both HG and LG groups made a significant gain in BOMAT performance after training (with no significant difference between HG and LG groups), suggesting that the amount of training gain on our complex spatial WM training task does not determine the magnitude of post-training G*f* improvement as measured by the BOMAT. To shed further light on the relationship between post-training gain and training task gain, as well as the influence of pre-training ability level, we conducted a series of multiple regression analyses for the BOMAT and each composite recognition memory measure incorporating these factors as explanatory variables. Interestingly, while training task gain, but not pre-training ability, predicted post-training improvement for the trainers’ average d′ and familiarity scores, the reverse was observed for the BOMAT and average recollection, with pre-training ability, but not training task gain, predicting post-training improvement. Although it is not clear from the current study why post-training BOMAT performance, recollection, familiarity and d′ were associated with different significant predictors, our data highlight that both training task gain and pre-training ability need to be taken into consideration when designing a cognitive training regime and that the nature of the influence of these factors can depend not only on the training task used, but also the cognitive tasks to which successful transfer is sought.

One point worth highlighting is that we used recognition tasks incorporating confidence judgments to assess EM, which may be more sensitive than standard neuropsychological EM tests or basic recognition and free recall paradigms. Broadly speaking, we found that all measures (d′, recollection, familiarity) benefited from successful training. The HG group’s improvements in d′, recollection and familiarity suggest that, despite an accumulation of evidence indicating that different MTL structures may subserve distinct EM processes [22], [23], or process different types of information [48], [49], training on a spatial WM task may lead to a non-specific improvement in EM, perhaps as a result of recruiting both PFC- and MTL-dependent processes. More specifically, in light of existing evidence arguing for a role for the hippocampus in recollection but not familiarity [22], and data suggesting that this structure is particularly important for spatial memory and processing [50], [51], one may have expected our complex spatial WM training task to predominantly benefit recollection memory and/or recognition memory for scenes. In contrast, we observed improvement to both recollection and familiarity, for both scene and object stimuli. Further research incorporating other types of EM tests (e.g. tasks involving other types of stimuli such as verbal material, recall paradigms, autobiographical memory, etc) will be necessary to determine whether the current findings can be generalized to all forms of EM.

It is important to note that, similar to a number of recent studies [12], [14], [25], [41], the current study used a non-active control group. It is plausible, therefore, that the transfer effects seen in the trainers may be explained by a difference in motivation between the trainers and the controls. In particular, a greater level of motivation in the trainers may have led to improved BOMAT and recognition memory performance in the trainers. Although this explanation cannot be ruled out entirely without additional research, there are indications that a difference in motivation may not account fully for our findings. First, only the HG, and not LG, group showed a significant improvement in both G*f* and EM. Since both of these groups underwent training and showed significant improvement on the training task (with the HG group showing the greatest gain) as well as the BOMAT, it is possible that the observed transfer effects were intervention-related. Second, our study involved young, highly educated participants for which differential motivation effects are likely to be less of an issue in comparison to other participant populations (e.g. brain damaged patients or elderly populations). Finally, recent work has demonstrated that there may not necessarily be significant differences in the use of passive and active control groups [52] suggesting, therefore, that although the use of an active control group may be optimal in cognitive training studies, the use of passive controls can be sufficient.

In conclusion, we have designed a spatial WM training paradigm that can potentially improve both EM and G*f*. Our findings are important not only because they reveal that WM training can benefit EM as measured by recognition memory, but also because they demonstrate that training on multiple tasks may not be necessary to produce performance gains in more than one cognitive domain. For training on a single task to transfer successfully to multiple abilities, it is crucial that careful consideration is given to the processes and types of stimuli that are involved in the training task in order to place a significant demand on overlapping cognitive processes and underlying neural correlates. Additional research will be necessary to determine the applicability of our findings to ageing and patient populations, and investigate whether improvements in EM can be observed beyond the scene and object recognition tasks used in the present study.

## References

[1] Craik FI, Rose NS (2012) Memory encoding and aging: A neurocognitive perspective. Neurosci Biobehav Rev 36: 1729–1739. 22155274 10.1016/j.neubiorev.2011.11.007

[2] Dickerson BC, Eichenbaum H (2010) The episodic memory system: neurocircuitry and disorders. Neuropsychopharmacology 35: 86–104. 19776728 10.1038/npp.2009.126 PMC2882963

[3] Hodges JR (2000) Memory in the dementias. In: Tulving E, Craik FIM, editors. The Oxford Handbook of Memory. New York, USA: Oxford University Press.

[4] Hornberger M, Piguet O (2012) Episodic memory in frontotemporal dementia: a critical review. Brain 135: 678–692. 22366790 10.1093/brain/aws011

[5] Vakil E (2005) The effect of moderate to severe traumatic brain injury (TBI) on different aspects of memory: a selective review. J Clin Exp Neuropsychol 27: 977–1021. 16207622 10.1080/13803390490919245

[6] Ranganath C, Flegal K, Kelly L (2011) Can cognitive training improve episodic memory? Neuron 72: 688–691. 22153366 10.1016/j.neuron.2011.10.022 PMC3894118

[7] Dahlin E, Neely AS, Larsson A, Backman L, Nyberg L (2008) Transfer of learning after updating training mediated by the striatum. Science 320: 1510–1512. 18556560 10.1126/science.1155466

[8] Simons JS, Spiers HJ (2003) Prefrontal and medial temporal lobe interactions in long-term memory. Nat Rev Neurosci 4: 637–648. 12894239 10.1038/nrn1178

[9] Burgess N, Hitch G (2005) Computational models of working memory: putting long-term memory into context. Trends Cog Sci 9: 535–541. 10.1016/j.tics.2005.09.011 16213782

[10] Owen AM, Hampshire A, Grahn JA, Stenton R, Dajani S, et al (2010) Putting brain training to the test. Nature 465: 775–778. 20407435 10.1038/nature09042 PMC2884087

[11] Buschkuehl M, Jaeggi SM, Hutchison S, Perrig-Chiello P, Dapp C, et al (2008) Impact of working memory training on memory performance in old-old adults. Psychol Aging 23: 743–753. 19140646 10.1037/a0014342

[12] Dahlin E, Nyberg L, Backman L, Neely AS (2008) Plasticity of executive functioning in young and older adults: immediate training gains, transfer, and long-term maintenance. Psychol Aging 23: 720–730. 19140643 10.1037/a0014296

[13] Richmond LL, Morrison AB, Chein JM, Olson IR (2011) Working memory training and transfer in older adults. Psychol Aging 26: 813–822. 21707176 10.1037/a0023631

[14] Schmiedek F, Lovden M, Lindenberger U (2010) Hundred Days of Cognitive Training Enhance Broad Cognitive Abilities in Adulthood: Findings from the COGITO Study. Front Aging Neurosci 2: 27. 20725526 10.3389/fnagi.2010.00027 PMC2914582

[15] Brehmer Y, Westerberg H, Backman L (2012) Working-memory training in younger and older adults: training gains, transfer, and maintenance. Front Hum Neurosci 6: 63. 22470330 10.3389/fnhum.2012.00063 PMC3313479

[16] Squire LR, Wixted JT (2011) The cognitive neuroscience of human memory since H.M. Annu Rev Neurosci. 34: 259–288. 10.1146/annurev-neuro-061010-113720 PMC3192650 21456960

[17] Stern CE, Sherman SJ, Kirchhoff BA, Hasselmo ME (2001) Medial temporal and prefrontal contributions to working memory tasks with novel and familiar stimuli. Hippocampus 11: 337–346. 11530838 10.1002/hipo.1048

[18] Piekema C, Kessels RP, Mars RB, Petersson KM, Fernandez G (2006) The right hippocampus participates in short-term memory maintenance of object-location associations. Neuroimage 33: 374–382. 16904344 10.1016/j.neuroimage.2006.06.035

[19] Lee ACH, Rudebeck SR (2010) Investigating the interaction between spatial perception and working memory in the human medial temporal lobe. J Cogn Neurosci 22: 2823–2835. 19925184 10.1162/jocn.2009.21396 PMC2929461

[20] Hannula DE, Ranganath C (2008) Medial temporal lobe activity predicts successful relational memory binding. J Neurosci 28: 116–124. 18171929 10.1523/JNEUROSCI.3086-07.2008 PMC2748793

[21] Ranganath C, D'Esposito M (2001) Medial temporal lobe activity associated with active maintenance of novel information. Neuron 31: 865–873. 11567623 10.1016/s0896-6273(01)00411-1

[22] Brown MW, Aggleton JP (2001) Recognition memory: what are the roles of the perirhinal cortex and hippocampus? Nat Rev Neurosci 2: 51–61. 11253359 10.1038/35049064

[23] Eichenbaum H, Yonelinas AP, Ranganath C (2007) The medial temporal lobe and recognition memory. Annu Rev Neurosci 30: 123–152. 17417939 10.1146/annurev.neuro.30.051606.094328 PMC2064941

[24] Hossiep R, Turck D, Hasella M (1999) Bochumer Matrizentest. BOMAT - advanced - short version. Gottingen: Hogrefe.

[25] Jaeggi SM, Buschkuehl M, Jonides J, Perrig WJ (2008) Improving fluid intelligence with training on working memory. Proc Natl Acad Sci U S A 105: 6829–6833. 18443283 10.1073/pnas.0801268105 PMC2383929

[26] Nelson HE (1982) National Adult Reading Test: Test Manual. Windsor, Berks: NFER-Nelson.

[27] Beck AT, Brown GM, Steer RA (1996) Beck Depression Inventory II manual. San Antonio, TX: The Psychological Corporation.

[28] Yonelinas AP (1994) Receiver-operating characteristics in recognition memory: evidence for a dual-process model. J Exp Psychol Learn Mem Cogn 20: 1341–1354. 7983467 10.1037//0278-7393.20.6.1341

[29] Yonelinas AP (2001) Components of episodic memory: the contribution of recollection and familiarity. Philos Trans R Soc Lond B: Biol Sci 356: 1363–1374. 11571028 10.1098/rstb.2001.0939 PMC1088520

[30] Howell DC (2010) Statistical Methods for Psychology. Belmont: Cengage Wadsworth.

[31] Dobbins IG, Foley H, Schacter DL, Wagner AD (2002) Executive control during episodic retrieval: multiple prefrontal processes subserve source memory. Neuron 35: 989–996. 12372291 10.1016/s0896-6273(02)00858-9

[32] Fletcher PC, Henson RN (2001) Frontal lobes and human memory: insights from functional neuroimaging. Brain 124: 849–881. 11335690 10.1093/brain/124.5.849

[33] Lee ACH, Robbins TW, Owen AM (2000) Episodic memory meets working memory in the frontal lobe: functional neuroimaging studies of encoding and retrieval. Crit Rev Neurobiol 14: 165–197. 12645957

[34] Rajah MN, McIntosh AR (2006) Dissociating prefrontal contributions during a recency memory task. Neuropsychologia 44: 350–364. 16051283 10.1016/j.neuropsychologia.2005.06.003

[35] Bunting MF, Conway ARA, Heitz RP (2004) Individual differences in the fan effect and working memory capacity. J Mem Lang 51: 604–622.

[36] Ranganath C, Blumenfeld RS (2005) Doubts about double dissociations between short- and long-term memory. Trends Cogn Sci 9: 374–380. 16002324 10.1016/j.tics.2005.06.009

[37] Olson IR, Moore KS, Stark M, Chatterjee A (2006) Visual working memory is impaired when the medial temporal lobe is damaged. J Cogn Neurosci 18: 1087–1097. 16839283 10.1162/jocn.2006.18.7.1087

[38] Hartley T, Bird CM, Chan D, Cipolotti L, Husain M, et al (2007) The hippocampus is required for short-term topographical memory in humans. Hippocampus 17: 34–48. 17143905 10.1002/hipo.20240 PMC2677717

[39] Olson IR, Page K, Moore KS, Chatterjee A, Verfaellie M (2006) Working memory for conjunctions relies on the medial temporal lobe. J Neurosci 26: 4596–4601. 16641239 10.1523/JNEUROSCI.1923-05.2006 PMC1764465

[40] Ranganath C, Cohen MX, Brozinsky CJ (2005) Working memory maintenance contributes to long-term memory formation: neural and behavioral evidence. J Cogn Neurosci 17: 994–1010. 16102232 10.1162/0898929054475118

[41] Jaeggi SM, Studer-Luethi B, Buschkuehl M, Su YF, Jonides J, et al (2010) The relationship between n-back performance and matrix reasoning - implications for training and transfer. Intelligence 38: 625–635.

[42] Morrison AB, Chein JM (2011) Does working memory training work? The promise and challenges of enhancing cognition by training working memory. Psychon Bull Rev 18: 46–60. 21327348 10.3758/s13423-010-0034-0

[43] Bor D, Owen AM (2007) A common prefrontal-parietal network for mnemonic and mathematical recoding strategies within working memory. Cereb Cortex 17: 778–786. 16707737 10.1093/cercor/bhk035

[44] Duncan J (2006) EPS Mid-Career Award 2004: brain mechanisms of attention. Q J Exp Psychol (Colchester) 59: 2–27. 10.1080/17470210500260674 16556554

[45] Gray JR, Chabris CF, Braver TS (2003) Neural mechanisms of general fluid intelligence. Nat Neurosci 6: 316–322. 12592404 10.1038/nn1014

[46] Jaeggi SM, Buschkuehl M, Jonides J, Shah P (2011) Short- and long-term benefits of cognitive training. Proc Nat Acad Sci USA 108: 10081–10086. 21670271 10.1073/pnas.1103228108 PMC3121868

[47] Schweizer S, Hampshire A, Dalgleish T (2011) Extending brain-training to the affective domain: increasing cognitive and affective executive control through emotional working memory training. PloS One 6: e24372. 21949712 10.1371/journal.pone.0024372 PMC3176229

[48] Graham KS, Barense MD, Lee ACH (2010) Going beyond LTM in the MTL: a synthesis of neuropsychological and neuroimaging findings on the role of the medial temporal lobe in memory and perception. Neuropsychologia 48: 831–853. 20074580 10.1016/j.neuropsychologia.2010.01.001

[49] Murray EA, Bussey TJ, Saksida LM (2007) Visual perception and memory: a new view of medial temporal lobe function in primates and rodents. Annu Rev Neurosci 30: 99–122. 17417938 10.1146/annurev.neuro.29.051605.113046

[50] Bird CM, Burgess N (2008) The hippocampus and memory: insights from spatial processing. Nat Rev Neurosci 9: 182–194. 18270514 10.1038/nrn2335

[51] Lee ACH, Buckley MJ, Pegman SJ, Spiers H, Scahill VL, et al (2005) Specialisation in the medial temporal lobe for processing of objects and scenes. Hippocampus 15: 782–797. 16010661 10.1002/hipo.20101

[52] Thorell LB, Lindqvist S, Bergman Nutley S, Bohlin G, Klingberg T (2009) Training and transfer effects of executive functions in preschool children. Dev Sci 12: 106–113. 19120418 10.1111/j.1467-7687.2008.00745.x

